# Tumor necrosis factor-α enhances hyperbaric oxygen-induced visfatin expression via JNK pathway in human coronary arterial endothelial cells

**DOI:** 10.1186/1423-0127-18-27

**Published:** 2011-05-04

**Authors:** Bao-Wei Wang, Chiu-Mei Lin, Gong-Jhe Wu, Kou-Gi Shyu

**Affiliations:** 1 School of Medicine, Fu-Jen Catholic University, New Taipei City, Taiwan; 2 Division of Cardiology, Shin Kong Wu Ho-Su Memorial Hospital, Taipei, Taiwan; 3 Department of Emergency Medicien, Shin Kong Wu Ho-Su Memorial Hospital, Taipei, Taiwan; 4 Department of Anesthesiology, Shin Kong Wu Ho-Su Memorial Hospital, Taipei, Taiwan; 5 Graduate Institute of Clinical Medicine, Taipei Medical University, Taipei, Taiwan

## Abstract

**Background:**

Visfatin, a adipocytokine with insulin-mimetic effect, plays a role in endothelial angiogenesis. Hyperbaric oxygen (HBO) has been used in medical practice. However, the molecular mechanism of beneficial effects of HBO is poorly understood. We sought to investigate the cellular and molecular mechanisms of regulation of visfatin by HBO in human coronary arterial endothelial cells (CAECs).

**Methods:**

Human CAECs were exposed to 2.5 atmosphere absolute (ATA) of oxygen in a hyperbaric chamber. Western blot, real-time polymerase chain reaction, and promoter activity assay were performed. In vitro glucose uptake and tube formation was detected.

**Results:**

Visfatin protein (2.55-fold) and mRNA (2.53-fold) expression were significantly increased after exposure to 2.5 ATA HBO for 4 to 6 h. Addition of SP600125 and JNK siRNA 30 min before HBO inhibited the induction of visfatin protein. HBO also significantly increased DNA-protein binding activity of AP-1 and visfatin promoter activity. Addition of SP600125 and TNF-α monoclonal antibody 30 min before HBO abolished the DNA-protein binding activity and visfatin promoter activity induced by HBO. HBO significantly increased secretion of TNF-α from cultured human CAECs. Exogenous addition of TNF-α significantly increased visfatin protein expression while TNF-α antibody and TNF-α receptor antibody blocked the induction of visfatin protein expression induced by HBO. HBO increased glucose uptake in human CAECs as HBO and visfatin siRNA and TNF-α antibody attenuated the glucose uptake induced by HBO. HBO significantly increased the tube formation of human CAECs while visfatin siRNA, TNF-α antibody inhibited the tube formation induced by HBO.

**Conclusions:**

HBO activates visfatin expression in cultured human CAECs. HBO-induced visfatin is mediated by TNF-α and at least in part through JNK pathway.

## Background

Visceral fat accumulation has been shown to play crucial roles in the development of cardiovascular disease as well as the development of obesity-related disorders [1]. Recent evidences show that fat tissue is an active endocrine organ producing "adipocytokines", hormones that influence a diverse array of processes including appetite and energy balance, immunity, insulin sensitivity, haemostasis, blood pressure, lipid metabolism and angiogenesis, all factors which can impact cardiovascular disease [2]. The recently discovered adipocytokine, visfatin, also known as pre-B cell colony-enhancing factor, has been demonstrated to mimic the glucose-lowering effect of insulin and improve insulin sensitivity [3]. However, the effects of visfatin are not restricted to glucose homeostasis. Visfatin was upregulated by hypoxia in adipocytes and in breast cancer cell through hypoxia-inducible factor-1 [4,5]. Recently, visfatin was shown to play a role in endothelial angiogenesis by activation of fibroblast growth factor2, signal transducer and activator of transcription 3, and vascular endothelial growth factor and matrix metalloproteinase [6-9]. Several factors that could regulate visfatin synthesis have been identified [10,11]. Overall, visfatin is a cytokine with various functions [12].

Hyperbaric oxygen (HBO) therapy provides a significant increase in oxygen content in the hypoperfused tissue and the elevation in oxygen content in the hypoxic tissue induces powerful positive changes in ischemic repair process [13]. Therefore, HBO is successfully used for the treatment of a variety of clinical conditions [14]. HBO therapy promotes wound healing by directly enhancing fibroblastic replication, collagen synthesis, and the process of neovascularization in ischemic tissue [15].

Because of the emerging concept of coronary artery endothelial cells (CAECs) in the progress of angiogenesis and no data have been presented to verify the effect of HBO on the regulation of visfatin in human CAEC. Therefore we hypothesize that HBO activates a proinflammatory response mediated through a specific transcription factor, and downstream effects of this activation increased the expression of visfatin. Therefore, we sought to investigate the cellular and molecular mechanisms of regulation of visfatin by HBO in human CAECs. The induction of visfatin in human CAECs by HBO may elucidate the mechanisms responsible for the therapeutic effect of HBO.

## Methods

### Primary human coronary artery endothelial cells (CAECs) culture

Human coronary artery endothelial cells (CAECs) were originally obtained from PromoCell GmbH (Heidelberg, Germany). The cells were cultured in endothelial cell growth medium MV supplemented with 10% fetal bovine serum, 100 U/ml penicillin, and 100 μg/ml streptomycin at 37°C in a humidified atmosphere of 5% CO_2 _in air. Cells were grown to 80-90% confluence in 10 cm^2 ^culture dishes and were sub-cultured in the ratio of 1:2.

### HBO treatment

For HBO treatment, cells were exposed to 2.5 ATA (atmosphere absolute) of oxygen (98% oxygen plus 2% CO_2_) in a hyperbaric chamber for 2 to 8 h at 37°C. The small hyperbaric chamber was put in a temperature-controlled (37°C) incubator (Additional file 1, Figure S1). The oxygen tension was chosen based on the human treatment protocols [16]. For the inhibition of signal pathways, cells were pretreated with inhibitors for 30 min, and then exposed to HBO without changing medium. SP600125 (20 μM, CALBIOCHEM^®^, San Diego, CA) is a potent, cell-permeable, selective, and reversible inhibitor of c-Jun N-terminal kinase (JNK). SB203580 (3 μM, CALBIOCHEM^®^) is a highly specific, cell permeable inhibitor of p38 kinase. PD98059 (50 μM, CALBIOCHEM^®^) is a specific and potent inhibitor of extracellular-signal-regulated kinase (ERK) kinase. Wortmannin ((5 nM, Sigma Chemical, St. Louis, MO, USA) is a phosphatidylinositiol-3 (PI-3) kinase inhibitor.

### Western blot analysis

Cells under HBO were harvested by scraping and then centrifuged (300 × g) for 10 minutes at 4°C. The pellet was resuspended and homogenized in a Lysis Buffer (Promega Corp., Madison, WI, USA), centrifuging at 10,600 × g for 20 minutes. Bio-Rad Protein Assay was used for the measure of protein content. Equal amounts of protein (15 μg) were loaded into a 12.5% SDS-polyacrylamide minigel, followed by electrophoresis. Proteins were electroblotted onto nitrocellulose. The blots were incubated overnight in Tris-buffered saline containing 5% milk to block nonspecific binding of the antibody. Proteins of interest were revealed with specific antibodies as indicated (1:1000 dilution) for 1 hour at room temperature followed by incubation with a 1:5000 dilution of horseradish peroxidase-conjugated polyclonal anti-rabbit antibody for 1 h at room temperature. The membrane was then detected with an enhanced chemiluminescence detection system (ECL, Amersham, Buckinghamshire, England). Equal protein loading of the samples was further verified by staining mouse anti-tubulin monoclonal antibody from Santa Cruz Biotechnology Inc. All Western blots were quantified using densitometry.

### RNA isolation and reverse transcription

Total RNA was isolated from cells using the single-step acid guanidinium thiocyanate/phenol/chloroform extraction method. Total RNA (1 μg) was incubated with 200 U of Moloney-Murine Leukemia Virus reverse transcriptase in a buffer containing a final concentration of 50 mmol/L Tris-Cl (pH 8.3), 75 mmol/L KCl, 3 mmol/MgCl_2_, 20 U of RNase inhibitor, 1 μmol/L poly-dT oligomer, and 0.5 mmol/L of each dNTP in a final volume of 20 μL. The reaction mixture was incubated at 42°C for 1 h and then at 94°C for 5 minutes to inactivate the enzyme. A total of 80 μL of diethyl pyrocarbonate treated water was added to the reaction mixture before storage at -70°C.

### Real-time quantitative PCR

A Lightcycler (Roche Diagnostic s, Mannheim, Germany) was used for real -time PCR. cDNA was diluted 1 in 10 with nuclease-free water. 2 μL of the solution was used for the Lightcycler SYBR -Green mastermix (Roche Diagnostics): 0.5 μmol/L primer, 5 mmol/L magnesium chloride, and 2 μL Master SYBR-Green in nuclease-free water in a final volume of 20 μL. The initial denaturation phase for specific gene was 5 min at 95°C followed by an amplification phase as detailed below: denaturation at 95°C for 10 sec; annealing at 63°C for 7 sec; elongation at 72°C for 8 sec; detection at 79°C and for 45 cycles. Amplification, fluorescence detection, and post -processing calculation were performed using the Lightcycler apparatus. Individual PCR products were analyzed for DNA sequence to confirm the purity of the product.

### Promoter activity assay

Visfatin gene was amplified with forward primer, CCACCGACTCGTACAAG and reverse primer, GTGAGCCAGTAGCACTC. The amplified product was digested with MluI and BglII restriction enzymes and ligated into pGL3-basic luciferase plasmid vector (Promega) digested with the same enzymes. Site-specific mutations were confirmed by DNA sequencing. Plasmids were transfected into human CAECs using a low pressure-accelerated gene gun (Bioware Technologies, Taipei, Taiwan) essentially following the protocol from the manufacturer. Test plasmid at 2 μg and control plasmid (pGL4-Renilla luciferase) 0.02 μg was co-transfected with gene gun in each well, and then replaced by normal culture medium. Following 6 hours of HBO, cell extracts were prepared using Dual-Luciferase Reporter Assay System (Promega) and measured for dual luciferase activity by luminometer (Turner Designs).

### Electrophoretic mobility shift assay (EMSA)

Nuclear protein concentrations from cells were determined by Bio-rad protein assay. Consensus and control oligonucleotides (Santa Cruz Biotechnology Inc.) were labeled by polynucleotides kinase incorporation of [γ^32^P]-ATP. After the oligonucleotide was radiolabeled, the nuclear extracts (4 μg of protein in 2 μl of nuclear extract) were mixed with 20 pmol of the appropriate [γ^32^P]-ATP -labeled consensus or mutant oligonucleotide in a total volume of 20 μl for 30 min at room temperature. The samples were then resolved on a 4% polyacrylamide gel. Gels were dried and imaged by autoradiography. Controls were performed in each case with mutant oligonucleotides or cold oligonucleotides to compete with labeled sequences.

### Measurement of TNF-α concentration by enzyme-linked immunosorbent assay

Conditioned medium from human CAECs subjected to HBO and those from control cells were collected for TNF-α measurement. The level of TNF-α was measured by a quantitative sandwich enzyme immunoassay technique (Amersham Pharmacia Biotech, Buckinghamshire, England). The lowest limit of TNF-α ELISA kit was 5 pg/ml.

### Capillary-like network formation Assay

Capillary-like network formation was performed in an *in vitro *culture system. Matrigel 250 μL (BD Biosciences, MA) was coated in a 24-well culture plate and allowed to solidify (37°C, 1 hr). Human CAECs were cultured on a Matrigel matrix and were exposed to 2.5 ATA of oxygen (98% oxygen plus 2% CO_2_) in a hyperbaric chamber for 6 hrs at 37°C. After HBO treatment, cells were placed in a humidified incubator for 16 hrs with an atmosphere of 5% CO_2 _at 37°C. The capillary-like network formation was observed with a phase-contrast microscope (Nikon, Tokyo).

### Migration assay

The migration activity of human CAECs was determined using the growth factor-reduced Matrigel invasion system (Becton Dickinson) following the protocol provided by the manufacturer. 5 × 10^4 ^cells were seeded on top of ECMatrix gel (Chemicon International, Inc., Temecula, CA). Cells were then incubated at 37°C for 6 h with or without HBO. Three different phase-contrast microscopic high-power fields per well were photographed. The migratory cells with positive stain were counted and the observer was blind to the experiment.

### Glucose uptake in cultured human CAECs

Human CAECs were seeded on ViewPlate for 60 min (Packard Instrument Co., Meriden, CT) at a cell density of 5 × 10^3 ^cells/well in serum free medium for overnight. Recombinant human visfatin 100 ng/ml (AdipoGen, Inc., Incheon, Korea), visfatin siRNA, TNF-α antibody, or TNF-α was added to the medium. Glucose uptake was performed by adding 0.1 mmol/l 2-deoxy-D-glucose and 3.33 nCi/ml 2-[1,2-^3^H]-deoxy-D-glucose for various periods of time. Cells were washed with phosphate-buffered saline twice. Non-specific uptake was performed in the presence of 10 μM cytochalasin B and subtracted from all of the measured value. MicroScint-20 50 μl was added and the plate was read with TopCount (Packard Instrument Co., Meriden, CT). The radioactivity was counted and normalized to protein amount measured with a protein assay kit.

### Statistical analysis

The data were expressed as mean ± SD. Statistical significance was performed with analysis of variance (GraphPad Software Inc., San Diego, CA). Tukey-Kramer comparison test was used for pairwise comparisons between multiple groups after the ANOVA. A value of P < 0.05 was considered to denote statistical significance.

## Results

### HBO increases visfatin expression

**Figure 1:**
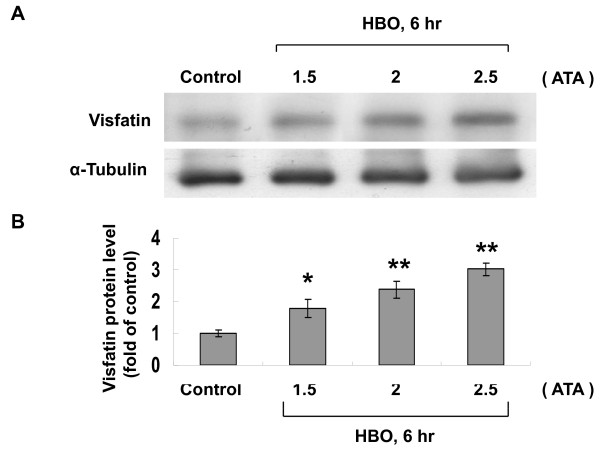
**Effect of HBO on visfatin protein expression**. A, Representative Western blot for visfatin in human CAECs treated with different degrees of HBO for 6 hour. B, Quantitative analysis of visfatin protein levels (n = 4 per group). *P < 0.05 vs. control. **P < 0.001 vs. control.

**Figure 2:**
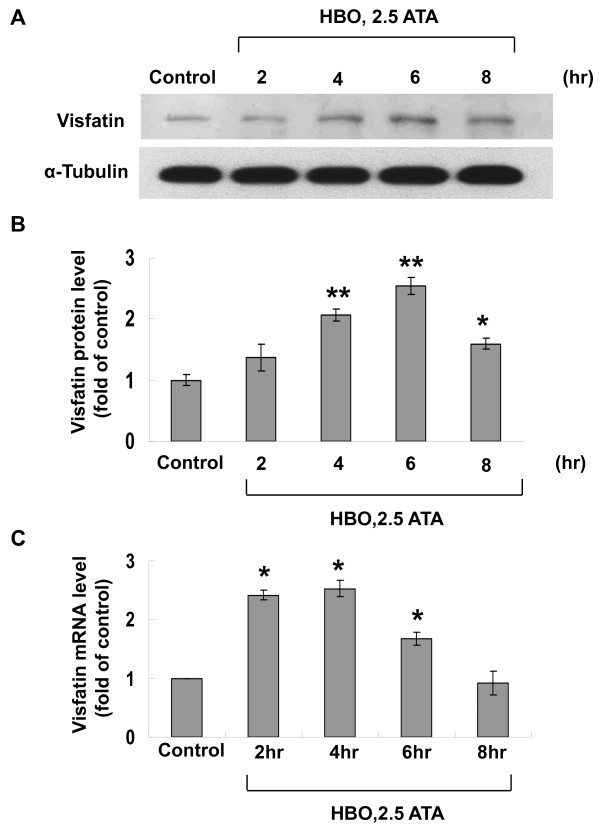
**HBO increases visfatin protein and mRNA expression in a time-dependent manner**. A, Representative Western blot for visfatin in human CAECs treated with various duration of HBO at 2.5 ATA. B, Quantitative analysis of visfatin protein levels (n = 4 per group). *P < 0.01 vs. control. **P < 0.001 vs. control. C, Quantitative analysis of visfatin mRNA levels. The values from treated human CAECs have been normalized to matched tubulin measurement and then expressed as a ratio of normalized values to mRNA in the control cells (n = 4 per group). *P < 0.01 vs. control.

To investigate the effect of HBO on the expression of visfatin protein, different degrees of ATA were used. As shown in Figure 1, the visfatin protein was significantly induced by HBO at 1.5, 2, and 2.5 ATA for 6 h. Since 2.5 ATA provided most powerful induction of visfatin protein. The following experiments used 2.5 ATA as the hyperbaric stimulation. The oxygen saturation measured by Oxy-Check (HANNA Instruments, Inc., Woonsocket, WI) and pO_2 _measured by pHOx Plus C (Nova Biomedical, Waltham, MA) in the medium was 523% and >800 mmHg, respectively after HBO treatment for 6 h and 77% and 175 mmHg, respectively in the control without HBO treatment. The levels of visfatin protein shown by Western blot analysis significantly increased at 4 and 6 h after HBO treatment (Figure 2A and 2B) as compared to control without treatment. Although visfatin protein level still maintained elevated after 8 h of HBO treatment, the level of visfatin protein tended to return to baseline level. Visfatin mRNA significantly increased at 2 h after HBO treatment, increased to maximal at 4 h and returned to baseline level at 8 h after HBO treatment (Figure 2C). Because visfatin protein was maximally induced at 6 h after HBO treatment, the following experiments were set for HBO treatment for 6 hours. To simulate the clinical application of HBO, HBO was also applied intermittently and repeatedly day by day at 1 h per day. The visfatin protein level increased by intermittent and repeat exposure was similar to that by 6 hr HBO exposure (Additional file 2, Figure S2)

### HBO-induced visfatin protein expression in human CAECs is mediated by JNK kinase

**Figure 3:**
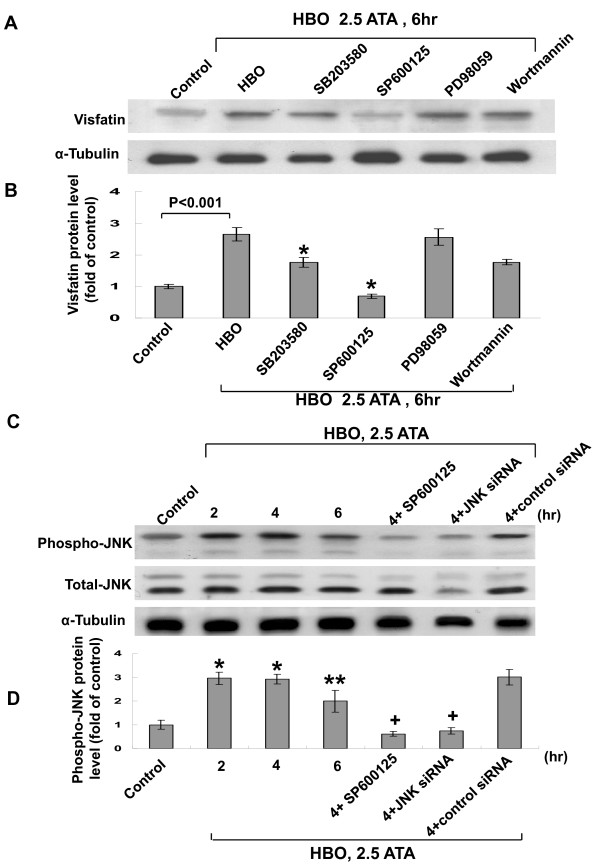
**HBO-induced visfatin expression in human CAECs is via JNK kinase**. A, Representative Western blots for visfatin protein levels in human CAECs subjected to HBO stimulation for 6 h in the absence or presence of inhibitors. B, Quantitative analysis of visfatin protein levels (n = 4 per group). *P < 0.001 vs. HBO. C, Representative Western blots for phosphor-JNK and total JNK protein levels in human CAECs subjected to HBO stimulation for 2 to 6 h in the absence or presence of inhibitor or siRNA. D, Quantitative analysis of phosphor-JNK protein levels (n = 4 per group). *P < 0.001 vs. control. **P < 0.01 vs. control. ^+^P < 0.001 vs. HBO at 4 h.

As shown in Figure 3A and 3B, the Western blot demonstrated that the HBO-induced increase of visfatin protein was significantly reduced after the addition of SB203580, and SP600125, 30 min before HBO treatment. The addition of PD98059 and wortmannin did not inhibit the visfatin protein expression induced by HBO. These findings implicated that JNK and ERK pathways but not p38 MAP kinase and PI-3 kinase mediated the induction of visfatin protein by HBO in human CAECs. Since JNK kinase inhibitor reduced the visfatin protein expression most significantly. We then focused on the JNK kinase pathway on the visfatin protein expression induced by HBO. HBO at 2.5ATA significantly increased the phosphorylation of JNK (Figure 3C and 3D). SP600125, inhibitor of JNK kinase, significantly attenuated the increased phosphorylation of JNK induced by HBO. JNK siRNA significantly attenuated the expression of phosphor-JNK induced by HBO. The scrambled siRNA did not affect the phosphorylation of JNK induced by HBO.

### HBO-induced visfatin protein expression in human CAECs is mediated by TNF-α

**Figure 4:**
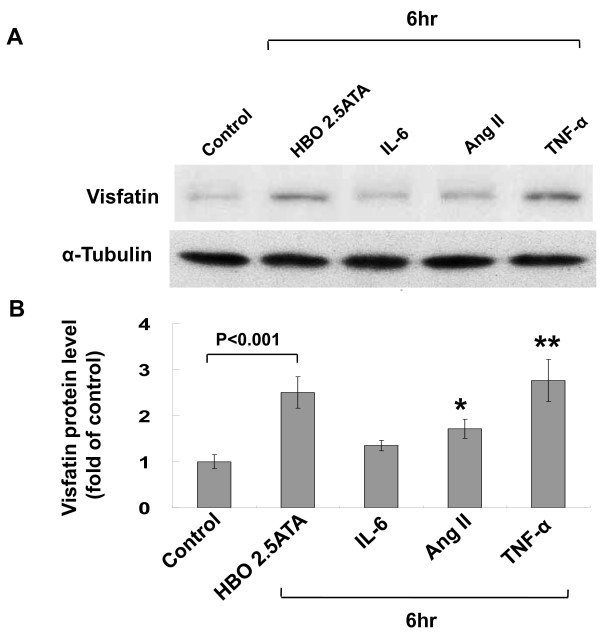
**Tumor necrosis factor-α (TNF-α) increases visfatin expression**. A, Representative Western blot for visfatin in human CAECs treated with different cytokines for 6 hour. B, Quantitative analysis of visfatin protein levels (n = 4 per group). *P < 0.05 vs. control. **P < 0.001 vs. control.

**Figure 5:**
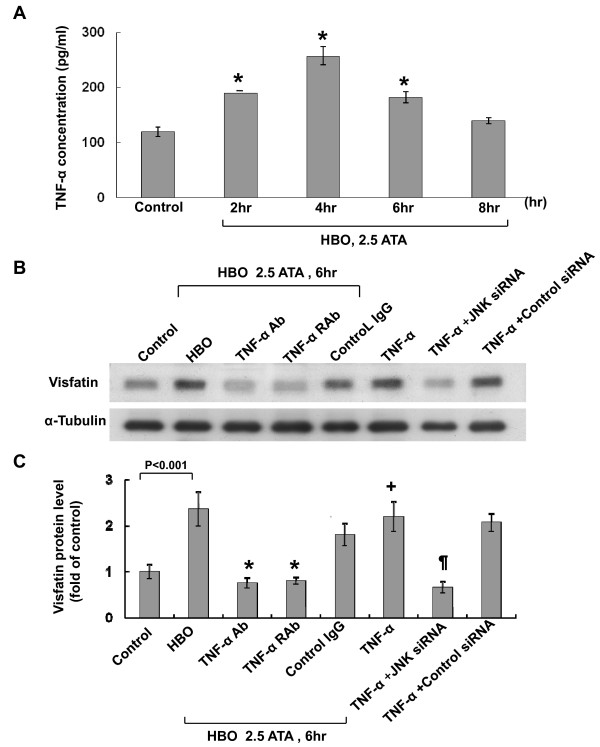
**TNF-α mediates the induction of visfatin by HBO**. A, HBO increased TNF-α secretion from human CAECs after HBO treatment. The secreted TNF-α was measured by ELISA method (n = 4 per group). *P < 0.01 vs. control. B, Representative Western blots for visfatin protein levels in human CAECs subjected to HBO stimulation for 6 h in the absence or presence of TNF-α, TNF-α monoclonal antibody, TNF-α receptor (TNF-α R) antibody and control IgG. (C) Quantitative analysis of visfatin protein levels (n = 4 per group). *P < 0.001 vs. HBO. ^+^P < 0.001 vs. control. ^¶^P < 0.001 vs. TNF-α.

Exogenous addition of TNF-α at 300 pg/ml significantly increased visfatin protein expression, similar to the level induced by HBO at 2.5 ATA (Figure 4). Exogenous addition of angiotensin II at 10 nM also increased visfatin expression but the increased level was less than that induced by TNF- α. Exogenous addition of IL-6 at 10 μg/ml did not increase visfatin expression. As shown in Figure 5A, HBO at 2.5 ATA significantly began to increase the TNF-α secretion from human CAECs at 2 h after HBO stimulation and remained elevated for 6 h and then returned to baseline level after 8 h. The HBO-induced vifatin protein expression was significantly attenuated by the addition of TNF-α antibody (5 μg/mL) or TNF-α receptor antibody (5 μg/mL). Addition of control IgG did not abolish the induction of visfatin protein by HBO. Exogenous addition of TNF-α at 300 pg/ml also induced the visfatin protein expression (Figure 5B and 5C). These data indicate that TNF-α mediates the induction of visfatin protein expression by HBO. JNK siRNA but not control siRNA significantly inhibited the visfatin expression induced by TNF-α

### HBO increases AP1-binding activity and visfatin promoter activity

**Figure 6:**
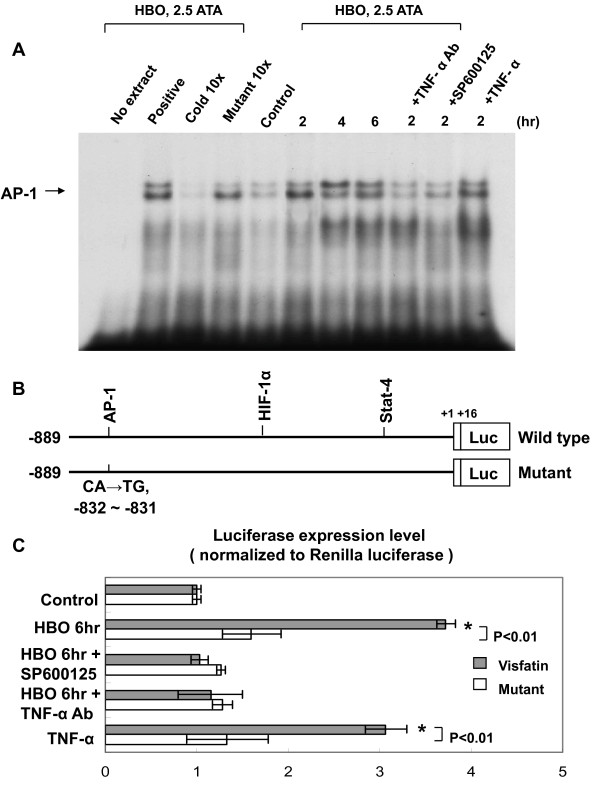
**HBO increases AP-1-binding activity and visfatin promoter activity**. A, Representative EMSA showing protein binding to the AP-1 oligonucleotide in nuclear extracts of human CAECs after HBO treatment in the presence or absence of inhibitors and TNF-α antibody. Similar results were found in another two independent experiments. Cold oligo means unlabeled AP-1 oligonucleotides. B, Constructs of visfatin promoter gene. Positive +1 demonstrates the initiation site for the visfatin transcription. Mutant visfatin promoter indicates mutation of AP-1 binding sites in the visfatin promoter as indicated. C, Quantitative analysis of visfatin promoter activity. The luciferase activity in cell lysates was measured and was normalized with renilla activity (n = 4 per group). *P < 0.001 vs. control.

Treatment of HBO for 2 h to 6 h significantly increased the DNA-protein binding activity of AP-1 (Figure 6A). An excess of unlabeled AP-1 oligonucleotide competed with the probe for binding AP-1 protein, whereas an oligonucleotide containing a 2-bp substitution in the AP-1 binding site did not compete for binding. Addition of SP600125 and TNF-α monoclonal antibody 30 min before HBO stimulation abolished the DNA-protein binding activity induced by HBO. Exogenous addition of TNF-α significantly increased the DNA-protein binding activity. To study whether the visfatin expression induced by HBO is regulated at the transcriptional level, we cloned the promoter region of human visfatin (-889~+16), and constructed a luciferase reporter plasmid (pGL3-Luc). The visfatin promoter construct contains AP-1, HIF-1α, and Stat-4 binding sites. As shown in Figure 6B and 6C, transient transfection experiment in human CAECs using this reporter gene revealed that HBO for 6 h significantly induced visfatin promoter activation. This result indicates that visfatin expression is induced at transcriptional level by HBO. When the AP-1 binding sites were mutated, the increased promoter activity induced by HBO was abolished. Moreover, addition of SP600125 and TNF-α antibody caused an inhibition of transcription. The increased promoter activity induced by exogenous addition of TNF-α was similar to that induced by HBO at 2.5 ATA. These results suggested that AP-1 binding site in the visfatin promoter is essential for the transcriptional regulation by HBO and that HBO regulates visfatin promoter via TNF-α and JNK pathways.

### Recombinant visfatin and HBO increase glucose uptake

**Figure 7:**
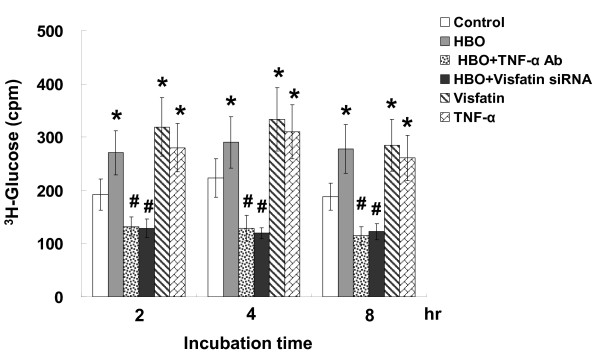
**Effect of HBO and recombinant visfatin on glucose uptake in human CAECs**. Glucose uptake was measured in human CAECs treated for 90 min with 100 ng/ml recombinant visfatin with or without siRNA, TNF-α, or TNF-α antibody (Ab). *P < 0.01 vs. control; ^#^P < 0.001 vs. HBO. Data are from 4 independent experiments.

HBO and recombinant human visfatin at 100 ng/ml significantly increased glucose uptake at various periods of incubation as compared to control human CAECs without treatment (Figure 7). The glucose uptake in HBO-treated cells was similar to that in exogenous addition of visfatin and TNF-α. Addition of visfatin siRNA or TNF-α antibody before HBO treatment attenuated the glucose uptake to baseline levels.

### HBO increases human CAECs tube formation and migration

**Figure 8:**
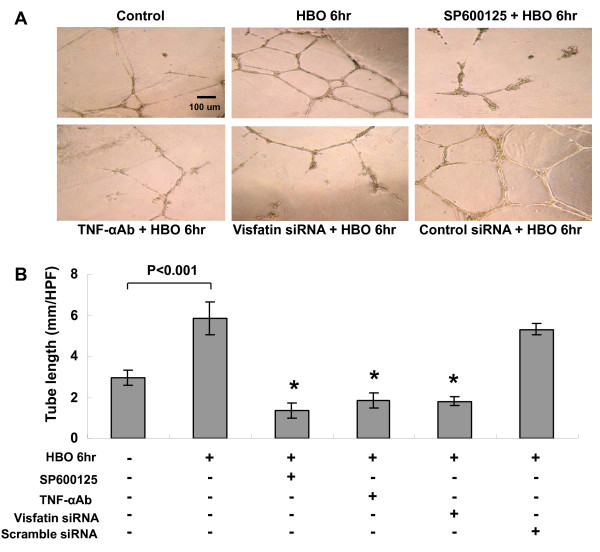
**Effect of HBO on human CAECs tube formation**. A, Representative image of tube formation of human CAECs. B, Quantitative tube length measurement (n = 5). *P < 0.001 vs. HBO alone.

**Figure 9:**
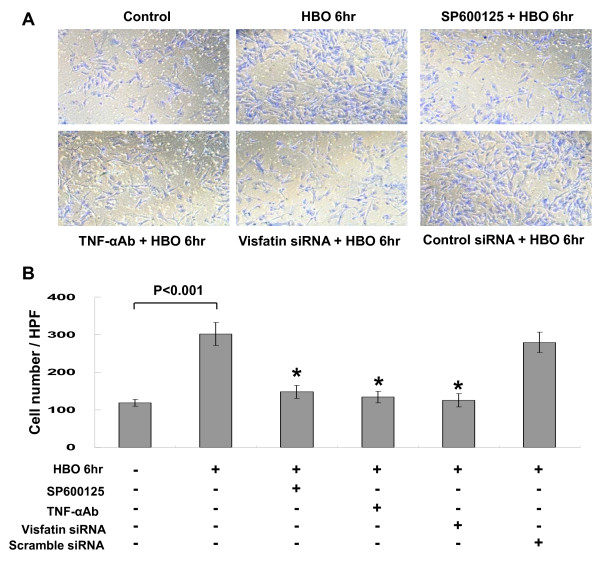
**Effect of HBO on human CAECs migration**. A, Representative image of migration of human CAECs. B, Quantitative migration activity measurement (n = 4). *P < 0.001 vs. HBO alone.

To test the effect of HBO on the function of human CAECs, tube formation and migration activity was examined. As shown in Figure 8, HBO for 6 h significantly increased the tube formation of human CAECs. Pretreatment with SP600125, TNF-α monoclonal antibody, and visfatin siRNA significantly blocked the induction of tube formation by HBO. The control siRNA did not inhibit the tube formation induced by HBO. HBO for 6 h significantly increased the migration activity of human CAECs (Figure 9). Pretreatment with SP600125, TNF-α monoclonal antibody, and visfatin siRNA significantly blocked the induction of migration by HBO. The control siRNA did not inhibit the migration induced by HBO.

## Discussion

In this study, we demonstrated several significant findings. Firstly, HBO induces transient visfatin expression in cultured human CAECs in a time- and load-dependent manner. Secondly, TNF-α acts as an autocrine factor to mediate HBO-induced visfatin protein expression in human CAECs. Thirdly, JNK kinase and AP-1 transcription factor are involved in the signaling pathways of visfatin induction by HBO. Fourthly, HBO increases tube formation and migration activity of human CAECs. The findings that HBO induces visfatin expression in human CAECs and functionally human CAECs tube formation and migration increase by HBO may further strengthen the effect of HBO on angiogenesis. In addition to insulin-mimetic effect, visfatin has been shown to induce angiogenesis via fibroblast growth factor, STAT-3 and vascular endothelial growth factor [6-8]. Recently, Adyaet al also reported that visfatin induces angiognesis via monocyte-chemoattractant protein-1 in human endothelial cells [17]. Our study confirmed the effect of angiogenesis of visfatin on human CAECs after HBO treatment.

Visfatin is a cytokine with various functions [12]. Recently, visfatin was shown to induce inflammatory cytokines expression in human endothelial cells and then caused endothelial dysfunction [18]. However, Lim et al. demonstrated cardio-protective effect of visfatin which is capable of reducing myocardial injury when administered at the time of myocardial reperfusion [19]. Our study demonstrated that HBO induced secretion of TNF-α, an inflammatory cytokine, from human CAECs and TNF-α increased visfatin expression by autocrine mechanism because exogenous administration of TNF-α also enhanced visfatin expression and TNF-α and TNF-α receptor antibody attenuated the increase of visfatin by HBO. AP-1 is a principal transcriptional factor that is activated by TNF-α and AP-1 is a well-characterized down-stream target of JNK. In this study, we demonstrated that HBO increased AP-1 binding activity. TNF-α antibody and SP600125 inhibited the AP-1 binding activity induced by HBO, indicating that TNF-α and JNK pathway mediate the increased transcriptional activity of AP-1 in the HBO model. Furthermore, TNF-α antibody and SP600125 attenuated the promoter activity of visfatin by HBO. Our data also indicate that AP-1 binding site in the visfatin promoter is essential for the transcriptional regulation by HBO because a mutant AP-1 binding site in the visfatin promoter abolished the transcriptional activity induced by HBO.

We have used three proinflammatory cytokines to stimulate human CAECs. Interleukin-6 did not have any effect on visfatin release and angiotensin II had partial effect on visfatin release. TNF-α produced most potent effect on visfatin release. Storka et al. have demonstrated no effect of angiotensin II on the release of visfatin from human umbilical vein endothelial cells, adipocytes and skeletal muscle cells [20]. The discrepancy result may indicate different cell types respond differently to pro-inflammatory cytokine stimulation to release visfatin.

Endothelial progenitor cell mobilization, homing and wound healing were enhanced by HBO through nitric oxide pathway [21-23]. However, the direct effect of HBO on human CAECs has not reported yet. In this study, we investigated the direct effect of HBO on human CAECs and demonstrated that HBO enhanced visfatin expression. HBO may induce different genes expression in different cell types via different signal pathways [24,25]. Hyperoxia with 100% oxygen has been shown to generate reactive oxygen species and produce vasoconstriction of coronary artery [26]. HBO is different with hyperoxia because hyperoxia is administered with normal baric pressure. The use of hyperbaric pressure of oxygen makes HBO a safe and noninvasive modality for the treatment of many kinds of diseases. In this study, HBO induces tube formation of human CAECs, an essential part for angiogenesis, indicating that HBO may be applied for patients with refractory ischemic heart diseases with non-optional therapy. In the study, HBO also significantly increased formation of reactive oxygen species for 2 to 8 h (Additional file 3, Figure S3). This result may suggest that HBO for 2-8 h causes oxygen toxicity.

In the present study, a protocol of 2.5 ATA 6 h HBO is definitely not a "treatment" protocol. In the clinical treatment protocol, HBO is applied intermittently and repeatedly day by day at 1 h per day. Prolonged exposure to HBO causes significantly toxicity effects to the neurologic system as well as to any cultured cells. The visfatin induced by HBO in our study protocol may be caused by oxygen toxicity. In the present study, we found that HBO for 6 h increased human CAECs migration and proliferation, an early process of angiogenesis. Exogenous addition of visfatin also increased migration of human CAECs without HBO stimulation (Additional file 4, Figure S4). Visfatin siRNA attenuated the migration and proliferation of human CAECs induced by HBO. MTT assay did not show significant cytotoxicity of HBO on human CAECs as compared to control or visfatin treatment (Additional file 4, Figure S4). The increased visfatin induced by HBO to increase angiogenesis may counteract the oxygen toxicity by prolonged HBO exposure.

Visfatin has been demonstrated to mimic the glucose-lowering effect of insulin and improve insulin sensitivity [3]. In this study, we have demonstrated that HBO increases glucose uptake in human CAECs, similar to the effect of visfatin. Visfatin siRNA attenuated the glucose uptake by HBO. This finding indicates that visfatin mediates the glucose uptake by HBO in human CAECs. Glucose uptake increase by HBO may improve the energy metabolism in CAECs which may provide myocardial protection and anti-ischemic effect.

## Conclusions

Our study reports for the first time that HBO enhances visfatin expression in cultured human CAECs. The HBO-induced visfatin is mediated by TNF-α and at least in part through JNK pathway. Visfatin increases angiogenesis after HBO, indicating that visfatin counteracts the oxygen toxicity by HBO.

## List of abbreviations used

AP-1: activating protein-1; CAECs: coronary artery endothelial cells; ERK: extracellular-signal-regulated kinase; EMSA: electrophoretic mobility shift assay; HBO: hyperbaric oxygen; JNK: c-Jun N-terminal kinase; siRNA: small interfering RNA; TNF-α: tumor necrosis factor-α; TLR: toll-like receptor; TNF-α: tumor necrosis factor-α.

## Competing interests

The authors declare that they have no competing interests.

## Authors' contributions

BWW has participated in the design of the study and has made substantial contributions to conception and design, or acquisition of data, or analysis and interpretation of data. CML has made substantial contributions to conception and design, or acquisition of data, or analysis and interpretation of data. GJW has made substantial contributions to conception and design, or acquisition of data, or analysis and interpretation of data. KGS has participated in the design of the study and drafted the manuscript. All authors have read and approved the final manuscript

## Supplementary Material

Additional file 1 **Figure S1: Schematic diagram of hyperbaric chamber in incubator**. Click here for file

Additional file 2 **Figure S2: Effect of intermittent and repeat HBO exposure on visfatin protein expression**. HBO at 2.5 ATA was applied 1 h per day. A, Representative Western blot for visfatin in human CAECs treated with HBO at 1 h each day for different duration. B, Quantitative analysis of visfatin protein levels (n = 4 per group). *P < 0.05 vs. control. **P < 0.001 vs. control. Click here for file

Additional file 3 **Figure S3: Effect of HBO on reactive oxygen species (ROS) formation in human CAECs**. A, Representative microscopic image for ROS assay with (left panel) or without green fluorescence (right panel). B, Quantitative analysis of the positive fluorescent cells. Control group indicates normoxia group. (n = 4 per group). *P < 0.05 vs. control. **P < 0.01 vs. control. Click here for file

Additional file 4 **Figure S4: Effect of recombinant visfatin on migration and viability of human CAECs**. A, Representative image of migration of human CAECs. B, Quantitative migration activity measurement (n = 4). *P < 0.001 vs. control. C, Quantitative analysis of viability by MTT assay. Click here for file
