# Supplementary material for: Tumor necrosis factor-α enhances hyperbaric oxygen-induced visfatin expression via JNK pathway in human coronary arterial endothelial cells
Source: J Biomed Sci. 2011 May 4;18(1):27. doi: 10.1186/1423-0127-18-27 (PMC3113732; doi:10.1186/1423-0127-18-27)
Supplement: Additional file 1 — Figure S1: Schematic diagram of hyperbaric chamber in incubator. [file 1423-0127-18-27-S1.PPT]

## Slide 1
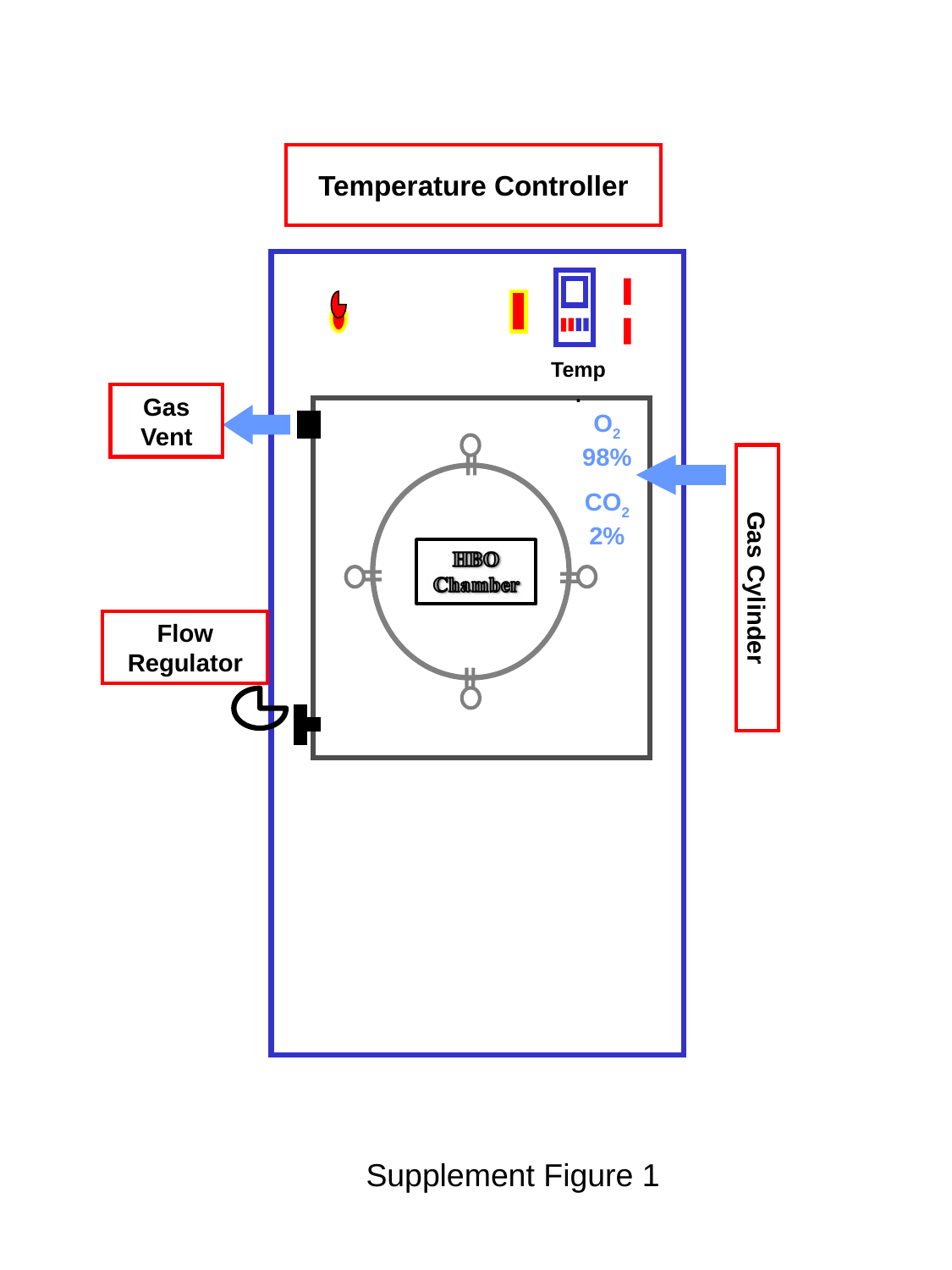

Temperature Controller
Temp.
Gas Vent
O2 98%
CO2 2%
Gas Cylinder
Flow Regulator
Supplement Figure 1
