# Supplementary material for: Tumor necrosis factor-α enhances hyperbaric oxygen-induced visfatin expression via JNK pathway in human coronary arterial endothelial cells
Source: J Biomed Sci. 2011 May 4;18(1):27. doi: 10.1186/1423-0127-18-27 (PMC3113732; doi:10.1186/1423-0127-18-27)
Supplement: Additional file 2 — Figure S2: Effect of intermittent and repeat HBO exposure on visfatin protein expression. HBO at 2.5 ATA was applied 1 h per day. A, Representative Western blot for visfatin in human CAECs treated with HBO at 1 h each day for different duration. B, Quantitative analysis of visfatin protein levels (n = 4 per group). *P < 0.05 vs. control. **P < 0.001 vs. control. [file 1423-0127-18-27-S2.PPT]

## Slide 1
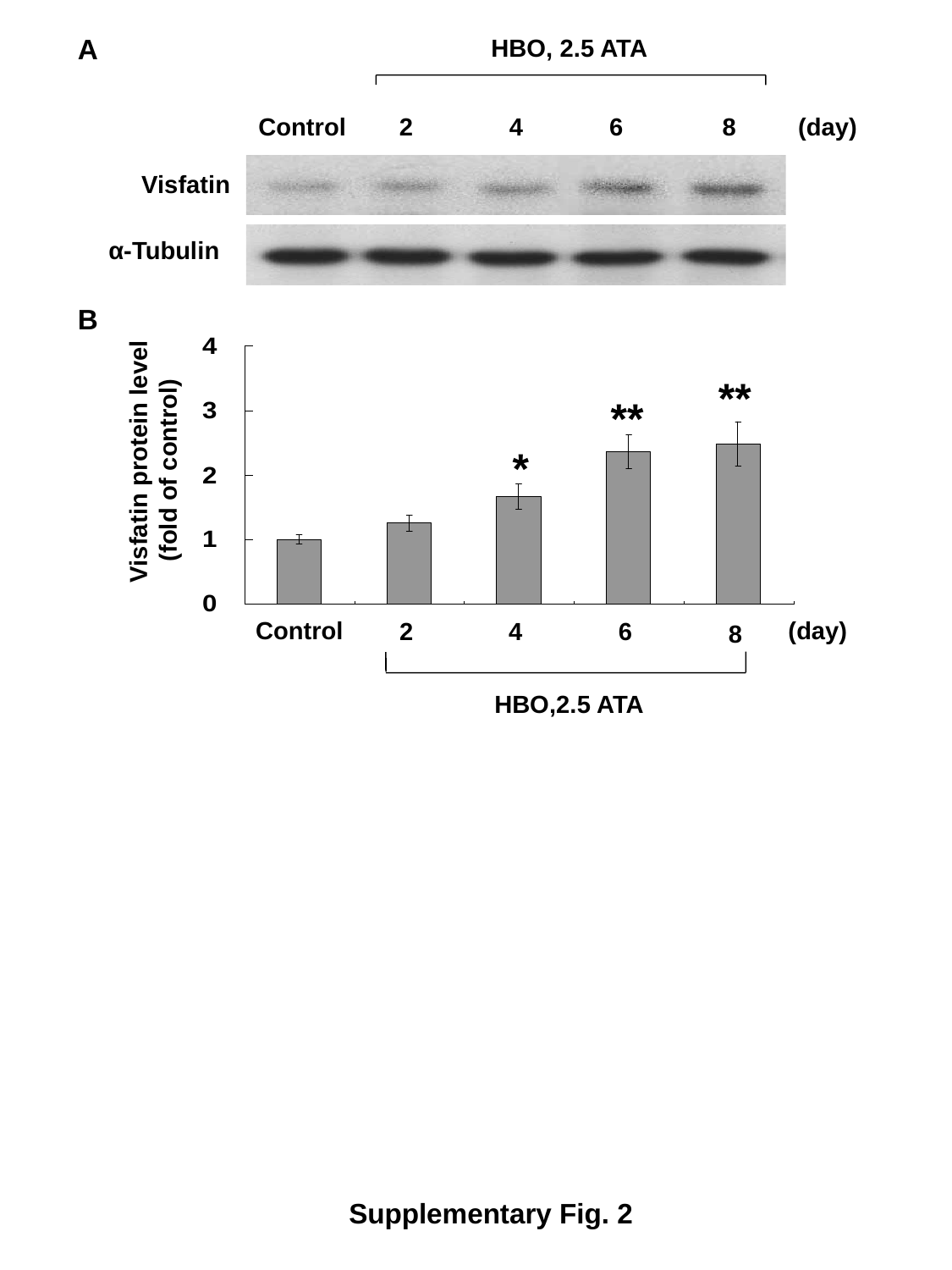

A
HBO, 2.5 ATA
Control
2
4
6
 8
(day)
Visfatin
α-Tubulin
B
**
**
Visfatin protein level
 (fold of control)
 *
Control
(day)
2
4
6
8
HBO,2.5 ATA
Supplementary Fig. 2
