# Supplementary material for: Tumor necrosis factor-α enhances hyperbaric oxygen-induced visfatin expression via JNK pathway in human coronary arterial endothelial cells
Source: J Biomed Sci. 2011 May 4;18(1):27. doi: 10.1186/1423-0127-18-27 (PMC3113732; doi:10.1186/1423-0127-18-27)
Supplement: Additional file 3 — Figure S3: Effect of HBO on reactive oxygen species (ROS) formation in human CAECs. A, Representative microscopic image for ROS assay with (left panel) or without green fluorescence (right panel). B, Quantitative analysis of the positive fluorescent cells. Control group indicates normoxia group. (n = 4 per group). *P < 0.05 vs. control. **P < 0.01 vs. control. [file 1423-0127-18-27-S3.PPT]

## Slide 1
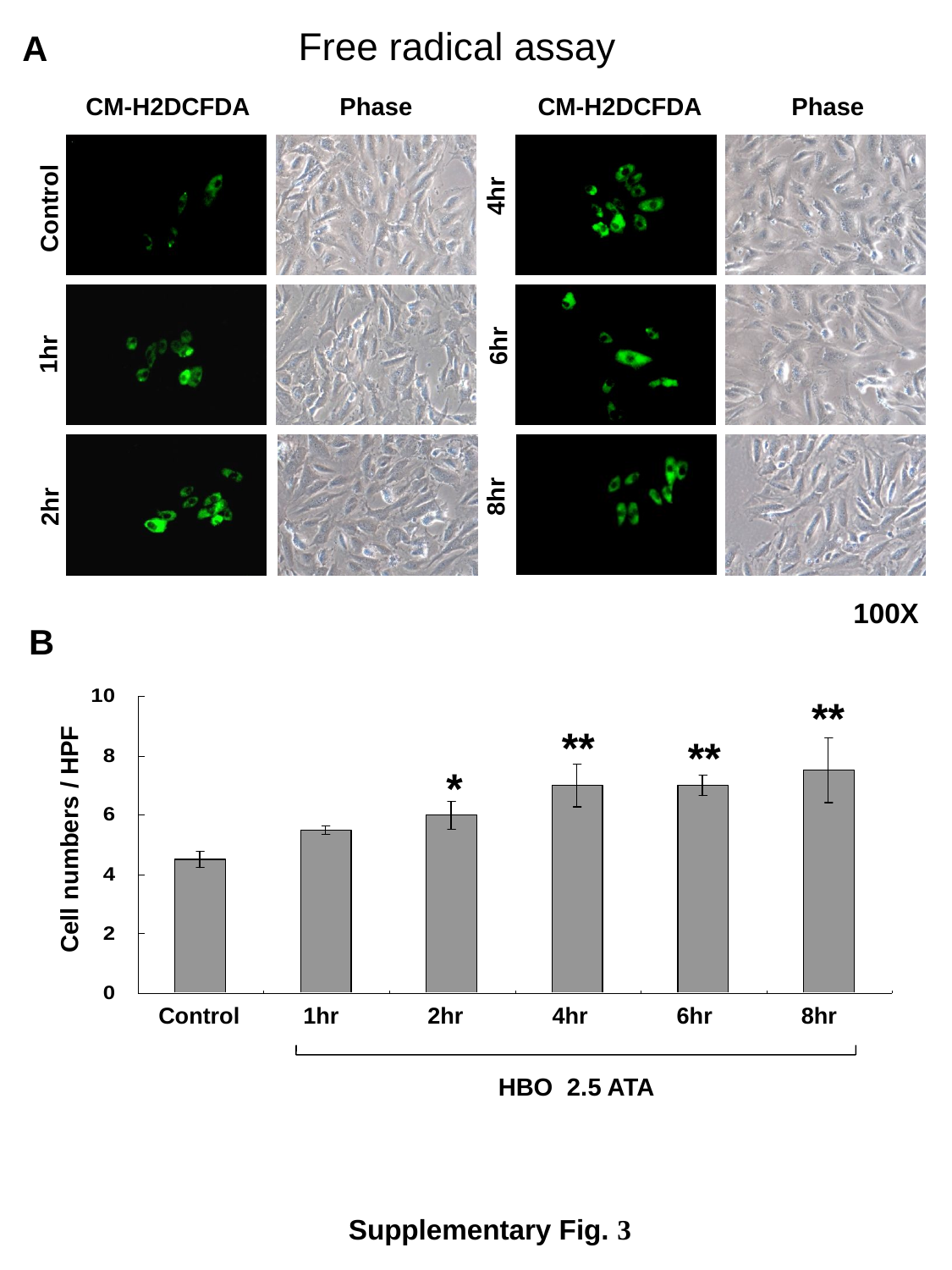

Free radical assay
A
CM-H2DCFDA Phase CM-H2DCFDA Phase
4hr
Control
6hr
1hr
2hr
8hr
100X
B
**
**
**
*
Cell numbers / HPF
Control 1hr 2hr 4hr 6hr 8hr
HBO 2.5 ATA
Supplementary Fig. 3
