# Supplementary material for: Tumor necrosis factor-α enhances hyperbaric oxygen-induced visfatin expression via JNK pathway in human coronary arterial endothelial cells
Source: J Biomed Sci. 2011 May 4;18(1):27. doi: 10.1186/1423-0127-18-27 (PMC3113732; doi:10.1186/1423-0127-18-27)
Supplement: Additional file 4 — Figure S4: Effect of recombinant visfatin on migration and viability of human CAECs. A, Representative image of migration of human CAECs. B, Quantitative migration activity measurement (n = 4). *P < 0.001 vs. control. C, Quantitative analysis of viability by MTT assay. [file 1423-0127-18-27-S4.PPT]

## Slide 1
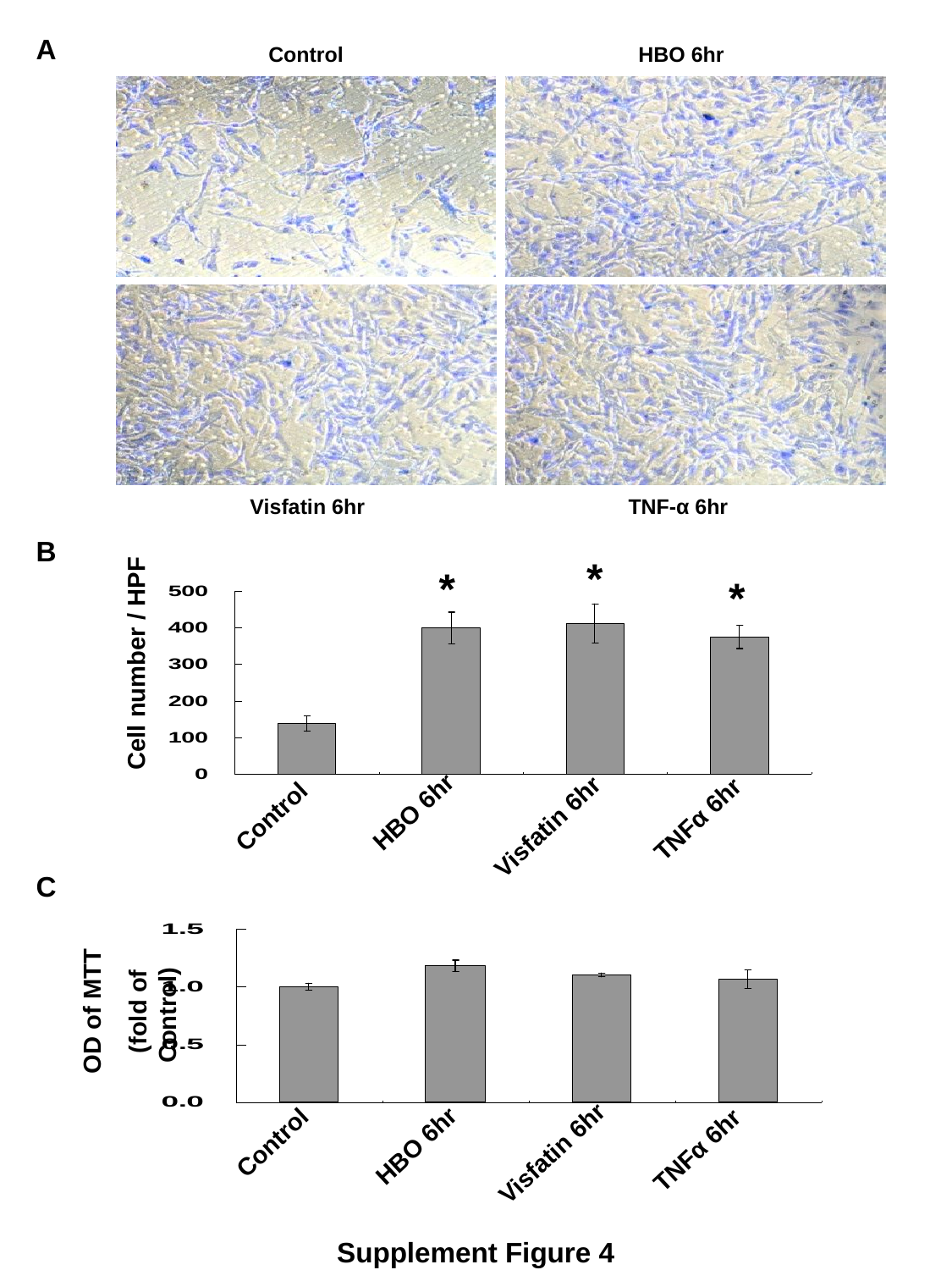

A
Control
HBO 6hr
Visfatin 6hr
TNF-α 6hr
B
*
*
*
Cell number / HPF
HBO 6hr
Control
TNFα 6hr
Visfatin 6hr
C
 OD of MTT
 (fold of Control)
Control
TNFα 6hr
HBO 6hr
Visfatin 6hr
Supplement Figure 4
